# The *Xenopus* model as a tool for investigating craniofacial developmental disorders

**DOI:** 10.3389/fmed.2025.1671687

**Published:** 2025-09-08

**Authors:** Qianying Kong, Huifang Peng, Qian Zhao, Hongwei Jiang, Xuechen Zhu

**Affiliations:** ^1^Henan Key Laboratory of Rare Diseases, Endocrinology and Metabolism Center, The First Affiliated Hospital, and College of Clinical Medicine of Henan University of Science and Technology, Luoyang, China; ^2^State Key Laboratory of Female Fertility Promotion, Department of Human Anatomy, Histology and Embryology, School of Basic Medical Sciences, Peking University Health Science Center, Beijing, China; ^3^Department of Pediatrics, Children's Medical Center, Peking University First Hospital, Beijing, China; ^4^Neuroscience Research Institute, Department of Neurobiology, School of Basic Medical Sciences, Key Laboratory for Neuroscience, Ministry of Education/National Health Commission of China, Peking University, Beijing, China

**Keywords:** *Xenopus*, craniofacial developmental disorders, CNCCs, disease model, gene editing, signaling pathway

## Abstract

Normal craniofacial development depends on the precise specification, migration, and differentiation of cranial neural crest cells (CNCCs). Perturbations in these processes result in a wide spectrum of congenital craniofacial anomalies, which represent a major cause of birth defects worldwide. *Xenopus* has emerged as a particularly powerful model for investigating craniofacial morphogenesis, owing to its external fertilization, large and experimentally accessible embryos, and evolutionarily conserved developmental pathways. These advantages allow direct *in vivo* visualization and manipulation of CNCCs behaviors at single-cell resolution, providing opportunities not readily achievable in mammalian models. With the integration of advanced techniques such as high-resolution imaging, lineage tracing, microsurgical manipulation, and genome editing, the utility of *Xenopus* in craniofacial biology has been greatly expanded. In this review, we outline the key stages of craniofacial development, summarize representative craniofacial developmental disorders studied using *Xenopus* as a model, and highlight how this system has provided critical mechanistic insights. Importantly, the amenability of *Xenopus* embryos to small-molecule screening underscores their translational potential as a rapid preclinical platform, linking human genetic variants to disease pathogenesis and accelerating therapeutic discovery for craniofacial disorders, as well as its translational potential as a rapid preclinical platform, linking human genetic variants to disease pathogenesis and accelerating therapeutic discovery for craniofacial disorders.

## 1 Introduction

Craniofacial morphogenesis is a tightly regulated and dynamic developmental process that governs the formation of head and facial structures through precise spatial and temporal patterning during embryogenesis. This process depends on a transient, pluripotent stem cell-like population known as neural crest cells (NCCs). Among these, CNCCs serve as key contributors to the regulation and execution of craniofacial morphogenesis (1). Any genetic or environmental perturbations affecting the induction, migration, proliferation, or fate determination of these cells may result in pronounced craniofacial developmental disorders (2).

It is estimated that congenital craniofacial developmental disorders represent about one-third of all congenital anomalies globally (3). Among these, cleft lip and/or palate (CL/P) is one of the most common, with a global incidence of around 1.7 per 1,000 live births (4). Another prominent condition, craniosynostosis, occurs at a rate of approximately 5.2 per 10,000 live births, with non-syndromic cases being the most frequent (5, 6). Craniofacial developmental disorders not only impact essential functions like feeding, breathing, and speech, but also impose substantial psychological stress on patients. The lifelong medical care, surgical interventions, and rehabilitative support required place a considerable burden on affected families and healthcare systems, underscoring the urgent need for deeper mechanistic understanding and preventive strategies.

Congenital craniofacial developmental disorders often arise from disruptions during early embryogenesis. To investigate the underlying mechanisms, a variety of model organisms have been employed, including the mouse (*Mus musculus*), chicken (*Gallus gallus*), zebrafish (*Danio rerio*), and the African clawed frog (*Xenopus laevis*). Among these, *Xenopus* stand out as a classical and highly tractable model system, offering several key advantages: external fertilization and development, high fecundity, a stable genetic background, and large, easily manipulable embryos. These features are particularly well-suited for live imaging, microsurgical manipulations, and microinjection-based gene perturbation, enabling precise analysis of early embryonic events and organogenesis (7). Importantly, *Xenopus* exhibits a high degree of genetic conservation with humans, sharing over 80% of known human disease-associated gene orthologs, including most genes implicated in craniofacial development, and its branchial arch (BA) structures are homologous to the pharyngeal arches (PA) that shape human facial development (8, 9). This evolutionary conservation, combined with its experimental accessibility, makes *Xenopus* a powerful and efficient model for dissecting the molecular and cellular mechanisms underlying congenital craniofacial disorders.

This review summarizes the key stages of craniofacial development and highlights recent advances using *Xenopus* models to study congenital craniofacial disorders. By integrating developmental genetics with functional modeling, *Xenopus* continues to offer critical insights into the etiology and potential treatment strategies for these complex conditions.

## 2 Key stages of craniofacial development

Craniofacial development is a temporally and spatially coordinated process that progresses through a series of well-defined stages, beginning at gastrulation and culminating in postnatal tissue remodeling. The *Xenopus* model provides a robust platform to investigate these stages *in vivo* due to its external development, accessibility for molecular manipulation, and conserved developmental pathways. Below, we summarize the key stages of craniofacial development, highlighting critical cellular events, regulatory signaling pathways, and clinically relevant disease associations (Table 1).

**Table 1:** Key stages of craniofacial development: developmental events, molecular regulators, and disease associations.

**Stage**	**Key developmental events**	**Major signaling pathways/factors**	**Representative disorders**
Gastrulation and germ layer formation	Formation of ectoderm, mesoderm, and endoderm via cell movements (invagination, epiboly, involution)	Nodal, BMP, Wnt	Holoprosencephaly (midline defects, cyclopia, cleft lip)
Neural plate border formation and NCC induction	Specification of NCCs at neural/non-neural ectoderm boundary; activation of neural crest specifiers	Pax3/7, *ZIC1*, Msx1/2; BMP, Wnt, FGF	Treacher Collins syndrome, CHARGE syndrome
NCC migration	EMT, delamination, and directional migration of CNCCs to craniofacial primordia	*SNAIL, SLUG, TWIST, SOX2 SOX10*; semaphorins, ephrins, chemokines	DiGeorge syndrome (*TBX1*), craniofacial and cardiovascular defects
Pharyngeal arch formation and patterning	Segmentation and patterning of arches; contribution to bone, cartilage, muscle, nerves	*HOX, TBX1, DLX; RA*, endothelin-1, FGF	*HOXA2* mutation (arch transformation), *TBX1*loss (DiGeorge syndrome)
Craniofacial skeletal morphogenesis	Intramembranous and endochondral ossification; cartilage and bone formation	*RUNX2, SOX9, COL2A1*	Craniosynostosis (*FGFR2, TWIST1, EFNB1* mutations)
Facial prominence fusion and palatogenesis	Growth and fusion of facial processes and palatal shelves	Shh, TGF-β3, *IRF6*	Cleft lip/palate, Van der Woude syndrome, Pierre Robin sequence
Postnatal growth and remodeling	Bone remodeling, suture maintenance, jaw shaping	Osteoblast/osteoclast regulation; hormonal, nutritional cues	Malocclusion, TMJ disorders, facial asymmetry

### 2.1 Gastrulation and germ layer formation

During gastrulation, occurring around stages 10–12 in *Xenopus*, the three primary germ layers—ectoderm, mesoderm, and endoderm—are formed through highly coordinated cellular movements such as invagination, involution, and epiboly (10). These germ layers serve as the embryonic source for all craniofacial tissues: the ectoderm gives rise to the neural crest and surface epithelium, the mesoderm contributes to vasculature and musculature, and the endoderm lines the pharyngeal foregut. Key signaling pathways involved in this process include Nodal, BMP, and Wnt, which establish the body axes and control cell fate decisions (11, 12). Disruptions in these early patterning signals can lead to severe craniofacial malformations. For example, defects in midline specification and forebrain patterning may result in holoprosencephaly, a condition often associated with facial dysmorphisms such as cyclopia, cleft lip, and nasal anomalies (13, 14).

### 2.2 Neural plate border formation and neural crest induction

**Figure 1:**
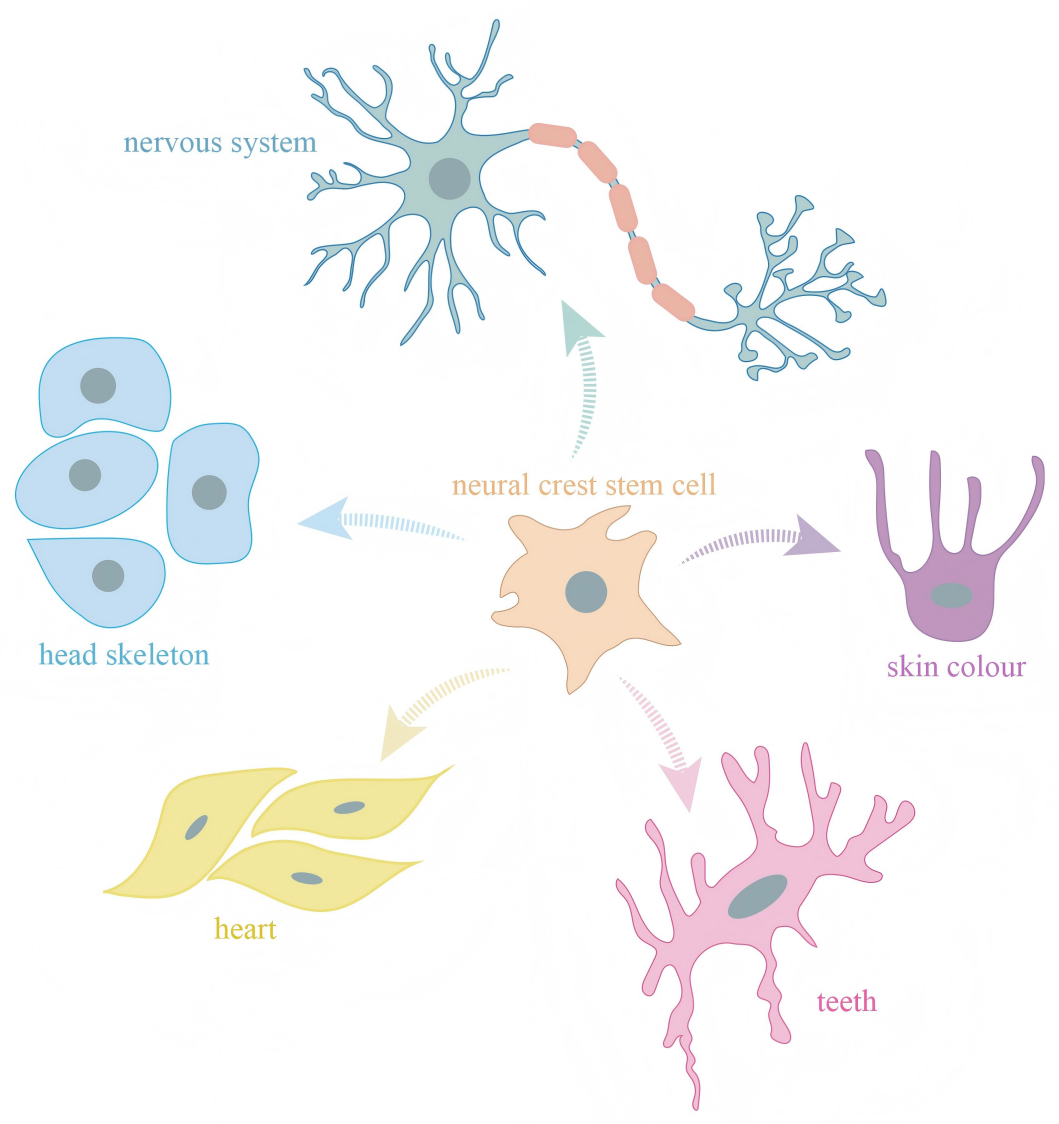
Neural crest cells possess multipotent differentiation potential and give rise to diverse cell types that contribute to a wide range of tissues and structures in vertebrates.

NCCs possess unique multipotency, giving rise to a diverse range of derivatives, including craniofacial bone and cartilage, peripheral neurons, glial cells, melanocytes, and various connective tissue types (Figure 1). NCCs are induced at the neural plate border, a region between the prospective neural and non-neural ectoderm. This induction is a result of a precise balance between BMP, Wnt, and FGF signaling gradients (15). Transcription factors such as Pax3/7, *ZIC1*, and Msx1/2 function as early neural crest specifiers, integrating these signals to define the neural crest territory (16). The induced neural crest progenitors then undergo further maturation and are primed for delamination and migration. Abnormal regulation of this process can impair neural crest formation, contributing to a spectrum of craniofacial syndromes. For instance, Treacher Collins syndrome arises from mutations affecting ribosome biogenesis in neural crest progenitors, whereas CHARGE syndrome involves mutations in *CHD7*, a chromatin remodeler essential for NCCs specification (17).

### 2.3 Neural crest cell migration

**Figure 2:**
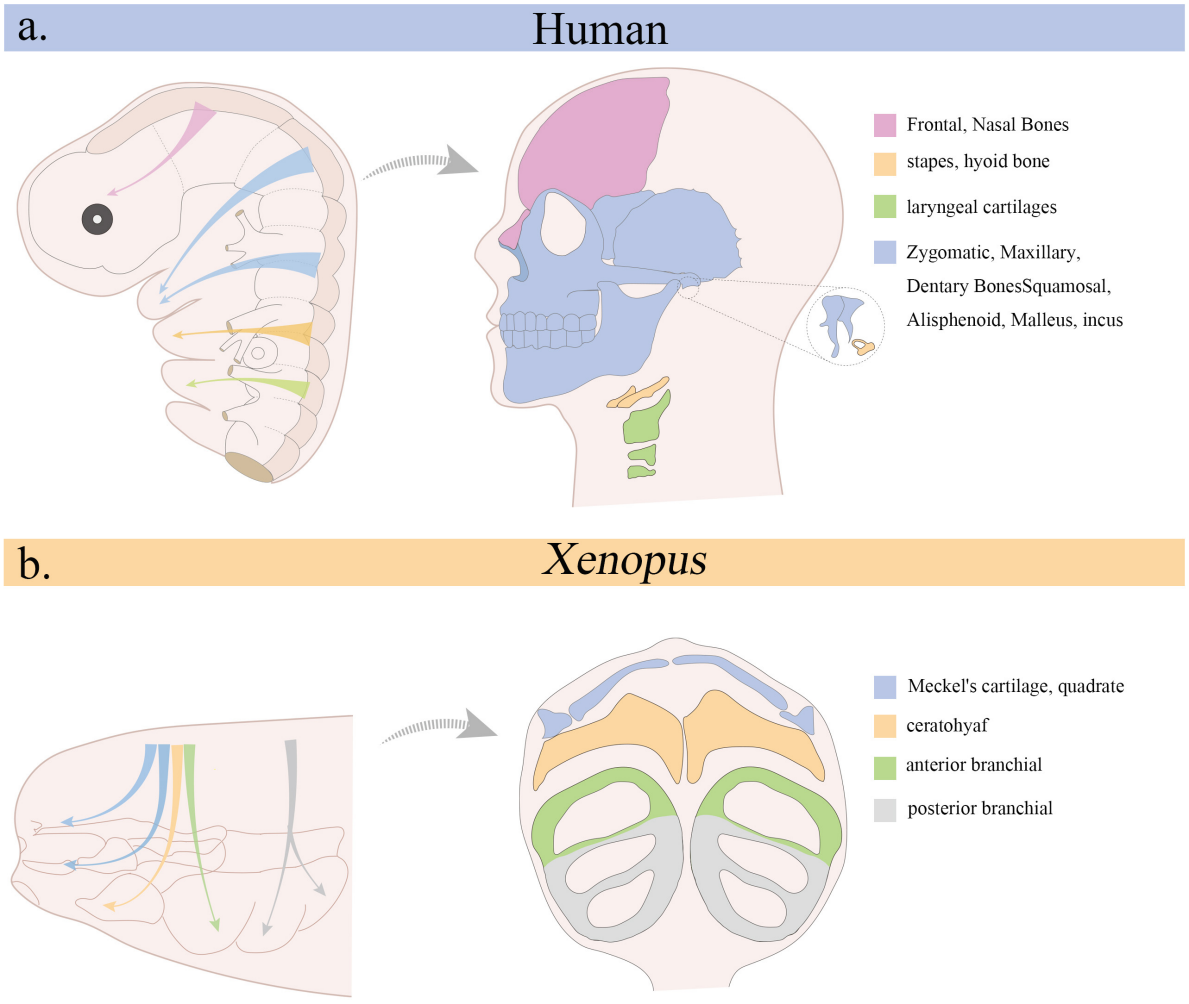
Migration of cranial neural crest and its contribution to cranial skeletal structures. **(a)** Origin of the cranial neural crest in the human embryo (left) and its derivatives in the adult head (right). **(b)** Migration pattern of cranial neural crest in *Xenopus* (left) and its contribution to cranial skeleton during the tadpole stage (right).

Following their specification, CNCCs undergo epithelial-to-mesenchymal transition (EMT), delaminate from the neural tube, and migrate along stereotypical pathways to populate the facial prominences and PA (Figure 2). This migration is tightly regulated by intrinsic transcriptional programs and extracellular cues. Key EMT regulators include *SNAIL, SLUG, TWIST*, and *SOX10*, which suppress epithelial traits and promote motility (18–20). In addition, *SOX2* modulates EMT during neural crest development, acting as a rheostat that fine-tunes the epithelial-to-mesenchymal transition and thereby influences cranial neural crest cell migration (21). Guidance signals, such as semaphorins, ephrins, and chemokines, help direct CNCCs to their appropriate destinations (22, 23). Defective migration or guidance can result in craniofacial malformations due to failed colonization of target tissues. DiGeorge syndrome, commonly caused by 22q11.2 deletions affecting *TBX1*, exemplifies how disrupted CNCCs migration can impair pharyngeal arch development, leading to mandibular hypoplasia, cleft palate, and cardiovascular defects (24).

### 2.4 Pharyngeal arch formation and patterning

The pharyngeal arches are transient, segmented structures composed of ectoderm, mesoderm, endoderm, and migrating CNCCs, which differentiate into diverse derivatives including bone, cartilage, nerves, and muscle. In *Xenopus*, the pharyngeal arches begin to form at stage 23 and are sequentially patterned along the anterior-posterior axis (25). Patterning is controlled by combinatorial expression of transcription factors such as *HOX, TBX1*, and the *DLX* family, along with signaling cues from RA, endothelin-1, and FGF (26–28). The first arch gives rise to the maxilla, mandible, and associated muscles and nerves, while the second arch contributes to the hyoid bone and facial musculature (29). Aberrant arch patterning leads to structural and functional anomalies in the craniofacial region. Mutations in *HOXA2* (30), for example, can cause duplication or transformation of arch derivatives, while defects in *TBX1* underlie many of the craniofacial and cardiovascular features in DiGeorge syndrome (31).

### 2.5 Craniofacial skeletal morphogenesis

Craniofacial skeletal development involves the differentiation of CNCCs-derived mesenchyme into cartilage and bone, guided by both genetic and environmental cues. Two primary modes of ossification are employed: intramembranous ossification, which forms flat bones such as the frontal and parietal bones of the skull, and endochondral ossification, which produces bones at the cranial base and parts of the pharyngeal skeleton (32, 33). Key transcription factors regulating this process include *RUNX2* for osteoblast differentiation, *SOX9* for chondrogenesis, and *COL2A1* as a cartilage matrix component (34–36). In *Xenopus*, these genes are expressed in a temporally and spatially conserved manner relative to mammals, making it an effective system for studying craniofacial skeletogenesis. Dysregulation in these pathways can lead to skeletal disorders such as craniosynostosis, characterized by the premature fusion of cranial sutures, often associated with mutations in *FGFR2, TWIST1*, or *EFNB1* (37).

### 2.6 Facial prominence fusion and palatogenesis

The vertebrate face forms through the coordinated growth and fusion of several facial prominences: the frontonasal, maxillary, and mandibular processes. Failure of these prominences to merge appropriately leads to cleft lip and/or palate. Palatogenesis involves the elevation, growth, and midline fusion of the palatal shelves, derived from the maxillary prominences (38). This process is intricately regulated by signaling molecules such as Shh (epithelial patterning), TGF-β3 (palatal shelf fusion), and *IRF6* (epithelial adhesion and remodeling) (39, 40). In *Xenopus*, palatal analog structures allow for the study of early patterning and epithelial-mesenchymal interactions relevant to human pathology. Mutations in TGF-β3 or *IRF6* result in cleft palate or syndromic forms of orofacial clefting such as Van der Woude syndrome and Pierre Robin sequence, highlighting the clinical relevance of this developmental stage (40, 41).

### 2.7 Postnatal growth and remodeling

Although *Xenopus* does not undergo mammalian-like postnatal growth, early developmental processes related to bone remodeling and jaw patterning provide insights into later craniofacial maturation (42). Craniofacial bones continue to grow and reshape through the coordinated actions of osteoblasts and osteoclasts, influenced by hormonal, nutritional, and mechanical stimuli. Remodeling is essential for accommodating dental eruption, reshaping the jaw for mastication, and aligning cranial sutures. Disruptions in these processes can lead to postnatal conditions such as malocclusion, temporomandibular joint disorders, and facial asymmetry (43). Studying early regulators of osteogenic and chondrogenic remodeling in *Xenopus* can inform our understanding of these pathologies in humans (44).

## 3 Model advantages of *Xenopus* in craniofacial research

Currently, commonly used model organisms for studying craniofacial development include the mouse, chick, zebrafish, and *Xenopus*. As a mammalian model, the mouse remains the gold standard for investigating the genetic underpinnings of craniofacial disorders due to its extensive genetic toolbox, including conditional knockouts and CRISPR/Cas9-based genome editing (45, 46). However, the *in utero* development of mouse embryos presents several limitations: a relatively long gestational period (~21 days), high maintenance and experimental costs, and technical challenges associated with real-time imaging. These factors reduce the efficiency of using mice for high-throughput screening or live imaging–based studies of early craniofacial development (47, 48) (Table 2).

**Table 2:** Comparative overview of vertebrate model organisms used in craniofacial developmental studies.

**Comparative dimensions**	**Mouse (*Mus musculus*)**	**Chicken (*Gallus gallus*)**	**Zebrafish (*Danio rerio)***	**African Clawed Frog (*Xenopus laevis*)**
Developmental mode	Embryonic development occurs *in utero*	Development occurs within the egg	External development with transparent embryos	External development with large, partially transparent embryos
Embryonic period	Approximately 21 days	Approximately 3 days (to completion of development)	Approximately 3 days	Approximately 2–4 days (morphological stages)
Gene editing tools	Cre/loxP, CRISPR (well-established systems)	Electroporation, RNAi; limited CRISPR application	Tol2 transposon, CRISPR, morpholino	CRISPR, morpholino, mRNA injection, transgenesis
Ease of embryonic manipulation	Difficult, requires cesarean section	Easy manipulation, suitable for electroporation and transplantation	Convenient for high-throughput injections	Extremely simple externalmicroinjection manipulation
Complexity of craniofacial structures	Complete (including secondary palate, cranial sutures, etc.)	Clearly defined pharyngeal arches and prominences	Simplified structures, lacking secondary palate	Developmental mechanisms of pharyngeal arches and prominences similar to mammals
Suitability for neural crest cell studies	Fate-mapping and lineage labeling well established	Easily tracked via electroporation	GFP labeling enables tracking	Established tracking systems such as Sox10-GFP
conservation of signaling pathways	BMP/Wnt/FGF/Shh conserved	Pathways conserved; widely studied expression patterns	Conserved expression; slightly lower collinearity	Strong pathway conservation; regulatory logic closely resembles humans
Limitations	High cost, long developmental cycle, and complex *in vivo* manipulation	Lack of gene knockout systems	Simplified anatomy, missing some craniofacial structures, partial lack of collinearity in regulation	Certain structures differ from humans (e.g., secondary palate); genetic resources less comprehensive than mouse
Representative applications	Human disease modeling, conditional mutation studies, knockout validation	Migration studies, electroporation, induction analysis	High-throughput drug screening, preliminary developmental pathway screening, mutation screening	Elucidation of early embryonic development, organogenesis processes, functional validation of signaling pathways
Major advantages	Well-established genetic tools, close relation to mammals	Intuitive manipulation, clear tissue localization	Low cost, rapid development, suitable for genetic screens	Real-time visualization, easy manipulation, high conservation of regulatory mechanisms

Chick embryos provide *ex utero* development, micromanipulation feasibility, and established developmental atlases (49–51). However, systematic genetic tools (e.g., conditional KO) remain limited (52, 53).

Zebrafish embryos are transparent, develop *ex utero*, and possess a well-characterized genetic background, making them highly suitable for high-throughput compound screening and live imaging–based analyses (54). With a mature and versatile neural crest cell labeling system, zebrafish offer unique advantages for investigating the early migratory phases of neural crest development (55). However, as a basal teleost species, zebrafish display a relatively simplified craniofacial anatomy and lack several mammalian-specific structures, such as the secondary palate, thereby limiting their capacity to fully recapitulate complex human craniofacial disease phenotypes (56). Moreover, the zebrafish genome has undergone extensive chromosomal rearrangements and transposon-driven expansions (57), resulting in a marked loss of synteny with many human genomic loci (58, 59). These structural alterations disrupt the spatial architecture between cis-regulatory elements (e.g., enhancers) and their target genes, complicating the conservation of transcriptional regulation. Consequently, gene regulatory elements in zebrafish often exhibit a more dispersed genomic organization and context-dependent activity, which can hinder efforts to model higher-order regulatory dynamics underlying human craniofacial disorders (60, 61).

Compared to zebrafish, *Xenopus*, as an amphibian model organism, offers an optimal balance between evolutionary conservation and experimental accessibility (62, 63). *Xenopus* exhibits a higher degree of conservation with mammals in terms of gene sequences, chromosomal synteny, cis-regulatory architecture, and transcriptional regulatory networks (64, 65). These features make *Xenopus* a powerful and versatile model for dissecting the genetic and molecular mechanisms underlying craniofacial development and associated disorders (64, 66). *Xenopus* is increasingly recognized as a critical phylogenetic bridge between lower vertebrate models and mammals, particularly with respect to genome architecture and the organization of impose substantial psychological stress on patients transcriptional regulatory networks (44, 65).

Additionally, invertebrate models such as *Drosophila melanogaster* and *Caenorhabditis elegans* have played significant roles in elucidating conserved developmental pathways, including Notch, Hedgehog, and Wnt signaling (67, 68). However, due to the absence of craniofacial structures, neural crest cells, and vertebrate-specific tissues such as bone and cartilage, these organisms cannot serve as direct models for studying craniofacial development and its associated disorders.

## 4 Advances in modeling craniofacial disorders using the *Xenopus* system

Compared with other vertebrate models, *Xenopus* uniquely combines close evolutionary proximity to mammals with high experimental accessibility, enabling direct *in vivo* visualization and manipulation of CNCCs development. Its external development and large embryos facilitate single-cell–level imaging of CNCCs specification, migration, and differentiation—advantages that murine models lack. While zebrafish excel in real-time neural crest tracking, their craniofacial complexity and regulatory architecture are less representative of mammals. Amphibians thus strike an optimal balance, retaining key mammalian craniofacial features and gene networks while allowing high-throughput genetic perturbation and microsurgical studies, making CNCCs biology particularly tractable in *Xenopus*.

Clinically, craniofacial disorders are broadly classified into two categories based on the presence or absence of associated systemic anomalies: non-syndromic craniofacial malformations, which occur in isolation, and syndromic craniofacial anomalies, which are accompanied by defects in other organ systems.

### 4.1 Non-syndromic craniofacial malformations

Non-syndromic craniofacial malformations are defined by isolated structural defects confined to the craniofacial region, without involvement of other organ systems. Representative conditions in this category include non-syndromic orofacial clefts (NSOC), non-syndromic craniosynostosis (NCS), and isolated microtia. NSOC can be further subdivided into non-syndromic cleft lip (NSCL), cleft lip with or without cleft palate (NSCLP), and cleft palate alone (NSCP). NSOC are among the most common congenital malformations, which can lead to feeding difficulties, speech and language delays, and other developmental challenges. Common clinical features include cleft lip and/or palate and associated functional impairments. The condition can be caused by pathogenic variants in *IRF6, RYK, TBX22, FGFR1, NAT2*, or *GSTT1*.

Studies utilizing the *Xenopus* model have significantly advanced our understanding of NSOC pathogenesis. Inhibition of the RA biosynthetic enzyme *RALDH2* or its receptor *RAR*γ induces clefting of the upper lip, suggesting that RA signaling modulates the expression of homeobox genes such as *LHX8* and *MSX2*, which are critical for boundary specification and tissue patterning during craniofacial development (69). Disrupting folate metabolism (via *dhfr* knockdown) combined with RA antagonism induces NSOC-like phenotypes. These include cleft lip, maxillofacial hypoplasia, and midfacial narrowing. Mechanistically, *dhfr* deficiency impairs cell proliferation, promotes DNA damage and apoptosis, and results in downregulation of *FGF8, RAR*γ, and *Wnt8*. Notably, folate supplementation partially rescues these defects, underscoring a functional interaction between folate metabolism and RA signaling (70).

Additional studies have identified *ISM1* as a crucial regulator of craniofacial morphogenesis. Loss of *ISM1* function leads to cleft lip/palate, abnormal development of the pharyngeal arches, reduced *lhx8* expression, and disruption of FGF8–Sprouty signaling, along with impaired expression of cell adhesion molecules such as integrin αvβ5. The identification of copy number variations and missense mutations in *ISM1* among human patients further supports its conserved role in orofacial development (71).

Together, these findings highlight the utility of the *Xenopus* model in uncovering the genetic and signaling mechanisms underlying non-syndromic orofacial clefts, and support its relevance for studying multifactorial and environmentally modulated craniofacial anomalies.

### 4.2 Syndromic craniofacial anomalies

This group of disorders features craniofacial malformations as a central component of complex syndromes, often accompanied by abnormalities in other organ systems. In *Xenopus* models, studies of such syndromic conditions can be broadly categorized based on the affected systems:

#### 4.2.1 Multiple craniofacial malformation syndromes

##### 4.2.1.1 Treacher collins syndrome

Treacher Collins Syndrome (TCS) is an autosomal dominant disorder. Features of TCS include microtia with conductive hearing loss, slanting palpebral fissures with possible coloboma of the lateral part of the lower eyelids, midface hypoplasia, micrognathia, as well as sporadically cleft palate and choanal atresia or stenosis. TCS is caused by pathogenic variants in the *TCOF1, POLR1D, POLR1C*, and *POLR1B* genes, with mutations in *TCOF1* accounting for more than 90% of cases (72, 73). The *TCOF1* gene codes for the nucleolar phosphoprotein Treacle (known as *xtreacle* in *Xenopus*), which is essential for ribosomal DNA transcription. Mutations in *TCOF1* lead to impaired rRNA synthesis, nucleolar stress, and apoptosis of neural crest cells (74, 75). In *Xenopus*, knockdown of *xtreacle* results in rDNA transcriptional defects consistent with the ribosome biogenesis abnormalities observed in human TCS patients. The resulting reduction in ribosome production mimics the nucleolar stress caused by *TCOF1* haploinsufficiency in TCS (76).

##### 4.2.1.2 Auriculocondylar syndrome

Auriculocondylar syndrome (ARCND) can be inherited in either an autosomal dominant or autosomal recessive manner. Features of ARCND include congenital ear clefts, mandibular condyle hypoplasia, temporomandibular joint abnormalities, micrognathia, small mouth, round facial appearance, and prominent cheeks. ARCND is caused by pathogenic variants in *GNAI3, PLCB4*, or *EDN1* (77–79). *Xenopus* models have demonstrated that both wild-type and ACS-mutant forms of *g*α*i3* can result in embryonic developmental defects. The mutant *g*α*i3*, which is unable to bind GTP, interferes with downstream signaling and disrupts neural crest differentiation by antagonizing *G*α*q* activity. These findings highlight a conserved cross-species mechanism underlying ACS pathogenesis (80).

#### 4.2.2 Multisystem syndromes

##### 4.2.2.1 Floating-Harbor syndrome

Floating-Harbor Syndrome (FHS) is an autosomal dominant disorder. Features of FHS include short stature, facial dysmorphism, delayed bone mineralization, speech impairment, and intellectual disability. FHS is caused by heterozygous truncating variants in the *SRCAP* gene (81, 82). A *Xenopus* model of FHS was generated using morpholino oligonucleotides targeting *srcap* to mimic human C-terminal truncating mutations, inducing typical craniofacial malformations. *SRCAP* mutations impaired CNCCs migration and downregulated key genes, such as *twist1* and *sox9*, suggesting neural crest dysfunction underlies the phenotype. The defects were rescued by wild-type *SRCAP* but not by the FHS-associated mutant. Mechanistically, *SRCAP* mutations disrupted nuclear localization, *H2A.Z.2* deposition, and AT-rich enhancer activity, highlighting the role of *H2A.Z.2* in neural crest development (8).

##### 4.2.2.2 CHARGE syndrome

CHARGE syndrome is an autosomal dominant disorder that presents with a broad range of congenital malformations affecting multiple organ systems. Common clinical features include external ear malformations, cranial nerve dysfunction (often with facial palsy), semicircular canal dysplasia or aplasia, choanal atresia, and craniofacial abnormalities such as cleft lip and/or palate. In addition, anosmia, genital hypoplasia, congenital heart defects, and tracheoesophageal anomalies are frequently observed. The syndrome is caused by heterozygous loss-of-function mutations in the *CHD7* gene (83). Patients often exhibit severe feeding difficulties, motor developmental delays, intellectual disabilities, and growth retardation. The majority of CHARGE syndrome cases result from haploinsufficiency of *CHD7*, a chromatin remodeling protein (84). In *Xenopus* embryos, knockdown of *chd7* leads to impaired expression of NCCs effector genes (e.g., *twist1, sox9*), disruption of its interaction with the PBAF complex, and aberrant pharyngeal arch development, recapitulating phenotypes such as choanal atresia. These effects are associated with dysregulation of guidance molecules such as *Sema3a* (85, 86).

##### 4.2.2.3 Axenfeld-Rieger syndrome

Axenfeld-Rieger Syndrome (ARS) is an autosomal dominant disorder. Features of ARS include anterior segment dysgenesis, glaucoma, dental anomalies, craniofacial dysmorphism, growth retardation, cardiovascular defects, redundant periumbilical skin, and pituitary defects leading to secondary endocrine disorders. ARS can be caused by pathogenic variants in the *PITX2, FOXC1*, and *FOXO1A* genes (87, 88). Studies using *Xenopus* models have demonstrated that *foxc1* expression is regulated by *VegT* via the Nodal signaling pathway. Loss of *foxc1* results in downregulation of adhesion molecules such as E-cadherin and the Ephrin/Eph system, causing mesodermal cell dissociation, axial shortening, and neural tube malformations. Injection of mutant *FOXC1* mRNA can partially rescue these phenotypes (89).

##### 4.2.2.4 Mowat-Wilson syndrome

Mowat-Wilson Syndrome (MWS) is an autosomal dominant disorder that presents with neural crest defects and distinctive craniofacial features, accompanied by a broad spectrum of multisystem abnormalities. Common clinical features include mild to severe intellectual disability, epilepsy, and congenital Hirschsprung disease. In addition, congenital malformations such as genital anomalies, congenital heart defects, corpus callosum agenesis, and ocular abnormalities are frequently observed. The syndrome results from heterozygous deletions or truncating mutations of the *ZFHX1B* gene (90). Patients typically exhibit a characteristic facial phenotype accompanied by a spectrum of multisystem abnormalities including mild to severe intellectual disability (ID), epilepsy, congenital Hirschsprung disease (HSCR), and frequently associated congenital malformations such as genital anomalies (most commonly hypospadias), congenital heart defects, corpus callosum agenesis, and ocular abnormalities (91). Knockdown of *mi-2*β in *Xenopus* reduces the expression of neural marker genes induced by *zfhx1b* and attenuates repression of BMP signaling pathway genes. Mutant *ZFHX1B* fails to effectively suppress BMP signaling, indicating that defective recruitment of the NuRD complex is a key pathogenic mechanism underlying craniofacial developmental defects (92).

##### 4.2.2.5 Cardiofaciocutaneous syndrome

Cardiofaciocutaneous syndrome (CFC) is an autosomal dominant disorder that presents with multisystem congenital anomalies and moderate to severe intellectual disability. Common clinical features include postnatal growth retardation with relative macrocephaly, characteristic craniofacial features such as prominent forehead, bitemporal narrowing, absent eyebrows, downslanting palpebral fissures with epicanthal folds, depressed nasal bridge, bulbous nasal tip, and distal limb anomalies. In addition, skin abnormalities such as dry, hyperkeratotic, scaly skin, sparse curly hair, and capillary malformations are frequently observed, along with congenital heart defects, most commonly pulmonary valve stenosis and hypertrophic cardiomyopathy. The syndrome is the result of heterozygous pathogenic variants in *BRAF, MAP2K1/MEK1, MAP2K2/MEK2, KRAS*, or *YWHAZ* (93). In *Xenopus* models, injection of the S230W variant of *YWHAZ* mRNA induces severe craniofacial malformations. Co-expression with Craf enhances GFP-Erk2 phosphorylation and rescues mesodermal defects, implicating sustained activation of the RAF–ERK signaling pathway in the pathogenesis (94).

##### 4.2.2.6 DDX3 syndrome

DDX3 syndrome is an X-linked dominant disorder that presents with neurodevelopmental delay, and intellectual disability, accompanied by a spectrum of multisystem abnormalities. Common clinical features include motor and language delay, autism spectrum disorder, epilepsy, and congenital brain and cardiac malformations (95), craniofacial anomalies are observed in the majority of affected individuals (96). The syndrome is the result of pathogenic variants in the *DDX3* gene. In *Xenopus* embryos, *ddx3* is specifically expressed at the neural plate border, pharyngeal arches (regions of neural crest cell migration), and head tissues. Knockdown of *ddx3* results in craniofacial cartilage hypoplasia, such as reduced size of the ceratohyal cartilage, along with downregulation of neural crest cell markers, indicating impaired neural crest induction (97).

##### 4.2.2.7 Fetal alcohol spectrum disorders

Fetal Alcohol Spectrum Disorders (FASD) affect 1%−5% of newborns in high-risk populations, characterized by smooth philtrum, thin vermilion border, and micrognathia. These craniofacial dysmorphisms correlate with neurocognitive deficits and require lifelong supportive care. Prenatal alcohol exposure is a critical factor causing FASD, which are characterized primarily by neurodevelopmental abnormalities and may be accompanied by craniofacial malformations, congenital organ defects, and growth retardation among other multisystem impairments (98). Exposure of *Xenopus* embryos to ethanol disrupts the expression of genes associated with later-stage NCCs migration. Supplementation with 5-methyltetrahydrofolate can partially rescue the observed phenotypes (99).

##### 4.2.2.8 Kabuki syndrome

Kabuki Syndrome (KS) is a rare neuro-developmental disorder caused by variants in genes of histone modification, including *KMT2D* and *KDM6A* (100) it is considered a Mendelian disorder of epigenetic regulation, affecting multiple organ systems including the nervous, sensory, immune, cardiac, renal, and skeletal systems, significantly impacting patients' quality of life (101). Knockdown of the *kmt2d* gene in *Xenopus* models recapitulates phenotypes of mandibular, hyoid, and pharyngeal arch cartilage hypoplasia. The underlying mechanism involves defects in CNCCs formation and migration, evidenced by downregulation of marker genes such as *twist*, abnormal cell dispersal capacity, and decreased levels of histone modifications H3K4me1 and H3K27ac. Further studies revealed that *kmt2d* regulates CNCCs migration by modulating the secreted factor *Sema3F*, and overexpression of *Sema3F* can partially rescue the migration defects (102).

##### 4.2.2.9 Wolf-Hirschhorn syndrome

Wolf-Hirschhorn syndrome (WHS) is araredisorderwithan estimated prevalence being around 1 in 50,000 births. The syndrome is caused by the deletion of a critical region (Wolf-Hirschhorn Syndrome Critical region—WHSCR) on chromosome 4p16.3. WHS is clinically characterized by pre-and postnatal growth restriction, hypotonia, intellectual disability, craniofacial dysmorphismand congenital fusion anomalies (103, 104), which are associated with aberrant development of CNCCs. Modeling this in *Xenopus* revealed that *whsc1/tacc3* knockdown disrupts neural crest migration, directly linking genetic loss to craniofacial pathogenesis (105).

##### 4.2.2.10 Musculocontractural Ehlers–Danlos syndrome

Musculocontractural Ehlers–Danlos syndrome (MC-EDS) is an autosomal recessive disease characterized primarily by increased connective tissue fragility, craniofacial structural abnormalities, congenital contractures, and impaired growth and development (106). It is caused by mutations in *DSE* or *CHST14*, which result in defects in craniofacial cartilage development. In *Xenopus* models, *dse* knockdown results in the downregulation of key EMT regulators in neural crest cells, including *snail2* and *twist1*, and impairs cell migration on fibronectin substrates, providing a cellular dynamic basis for the craniofacial anomalies associated with the disease (107).

##### 4.2.2.11 Branchio-oto-renal syndrome

Branchio-oto-renal (BOR) syndrome is an autosomal dominant disorder typically characterized by branchial cleft anomalies, ear developmental defects, and renal malformations. Additional clinical features may include abnormalities of the face, maxilla, ureters, and bladder, lacrimal system dysfunction, otitis media, and shoulder anomalies (108). About 40% of patients with BOS carry aberrations of *EYA1* gene which is the most important cause of BOS. A total of 240 kinds of pathogenic variations of *EYA1* have been reported in different populations so far, including frameshift, nonsense, missense, aberrant splicing, deletion and complex rearrangements (109). Knockdown of the *eya1* gene in *Xenopus* leads to defects in otic vesicle development (inner ear structural abnormalities) and hypoplasia of branchial arch cartilages, such as Meckel's cartilage, thereby recapitulating the craniofacial skeletal malformations observed in patients. Mechanistically, *eya1* deficiency downregulates neural crest cell marker genes, including *sox10* and *snail2*, suggesting that *eya1* regulates craniofacial development through modulation of neural crest cell migration and differentiation. Furthermore, wild-type human *EYA1* mRNA can partially rescue the phenotype (110, 111).

##### 4.2.2.12 Campomelic dysplasia

Campomelic Dysplasia (CD) is an autosomal dominant, perinatal lethal skeletal dysplasia characterized by a small chest and short long bones with bowing of the lower extremities. CD is the result of heterozygosity for mutations in the gene encoding the chondrogenesis master regulator, *SOX9* (112). It is caused by *SOX9* haploinsufficiency, manifesting as mandibular hypoplasia and craniosynostosis. Knockdown of *sox9* in *Xenopus* suppresses the expression of neural crest effector genes such as *snail2* and *foxd3*, resulting in complete loss of pharyngeal arch cartilages, including Meckel's cartilage, and malformation of the otic capsule, thereby recapitulating sensorineural hearing loss phenotypes (35).

##### 4.2.2.13 Oral-facial-digital syndrome

Oral-facial-digital (OFD) represent a heterogeneous group of rare developmental disorders affecting the mouth, the face and the digits. Additional signs may involve brain, kidneys and other organs thus better defining the different clinical subtypes. With the exception of OFD types I and VIII, which are X-linked, the majority of OFDS is transmitted as an autosomal recessive syndrome. A number of genes have already found to be mutated in OFDS and most of the encoded proteins are predicted or proven to be involved in primary cilia/basal body function (113). Injection of morpholino oligonucleotides targeting *ints13* into *Xenopus* embryos to knock down *ints13* expression results in developmental defects including microcephaly, shortened body axis, and tail curvature. Additionally, differentiation of multiciliated cells (MCCs) is impaired, accompanied by reduced cilia number and functional abnormalities, thereby recapitulating craniofacial hypoplasia phenotypes (114)

##### 4.2.2.14 Hamamy syndrome

Hamamy syndrome is a rare genetic disorder characterized by intellectual disability, sensorineural hearing loss, congenital cardiac anomalies with intraventricular conduction delay, hypopigmented microcytic anemia, and skeletal abnormalities of the long bones with recurrent fractures. Craniofacial features include severe hypertelorism and craniofacial skeletal dysmorphisms (115). Homozygous mutations in *IRX5* are associated with this syndrome. Knockdown of *irx5* in *Xenopus* disrupts the expression of the key chemokine *cxcl12* involved in cranial neural crest cell migration, impeding pharyngeal arch mesenchymal condensation. Moreover, knockdown of *cxcl12* partially rescues the phenotype, suggesting that *IRX5* regulates neural crest migration through negative modulation of chemotactic signaling (1).

##### 4.2.2.15 Nager syndrome

Nager syndrome is a rare human developmental disorder characterized by craniofacial defects including the downward slanting of the palpebral fissures, cleft palate, limb deformities, mandibular hypoplasia, hypoplasia or absence of thumbs, microretrognathia, and ankylosis of the temporomandibular joint. There is evidence of autosomal dominant and autosomal recessive inheritance for Nager syndrome, suggesting genetic heterogeneity. The majority of the described causes of Nager syndrome include pathogenic variants in the *SF3B4* gene, which encodes a component of the spliceosome; therefore, the syndrome belongs to the spliceosomopathy group of diseases (116). Knockdown of *sf3b4* in *Xenopus* results in downregulation of neural crest marker genes (*sox10, twist1*) and induces ectodermal apoptosis, suggesting that aberrant RNA splicing leads to craniofacial defects by impairing the survival of neural crest progenitor cells (117).

##### 4.2.2.16 Coronal craniosynostosis

Craniosynostosis is one of the most common congenital cranial malformations affecting approximately 6/10,000 live births (118), with increasing incidence trends reported over the recent decades (119). The condition, which is characterized by premature fusion of one or more cranial sutures, is classified according to the type (e.g., sagittal, coronal, lambdoid) and/or number (single/multiple) of affected sutures. Involvement of the coronal suture is relatively rare and accounts for only 11%−13% of all cases of single-suture craniosynostosis. Injection of mRNA encoding human *ZIC1* mutations (such as p. Ser388^*^ and p. Glu402) into *Xenopus* embryos enhances the expression of the downstream target gene *engrailed-2* (*en-2*), leading to aberrant neural crest signaling. Truncating variants, such as p. Ala437, retain the zinc finger domain and aberrantly activate *En-2*, thereby recapitulating craniosynostosis-like phenotypes in the model organism (120).

##### 4.2.2.17 Smith-Magenis syndrome

Smith-Magenis syndrome (SMS) is a complex genetic disorder characterized by distinctive physical features, developmental delay, cognitive impairment, and a typical behavioral phenotype. SMS is caused by interstitial 17p11.2 deletions (90%), encompassing multiple genes and including the retinoic acid-induced 1 gene (*RAI1*), or by pathogenic variants in *RAI1* itself (10%). *RAI1* is a dosage-sensitive gene expressed in many tissues and acting as transcriptional regulator. The majority of individuals exhibit a mild-to-moderate range of intellectual disability (121). Knockdown of *ra11* has been shown to reduce the expression of the neural crest cell marker *tfap2a*, leading to decreased craniofacial cartilage formation and increased apoptosis in the forebrain (122).

The Xenopus model has been employed to faithfully recapitulate a wide spectrum of craniofacial disease phenotypes—including cartilage hypoplasia, craniosynostosis, and hypertelorism—thus enabling precise *in vivo* and *in vitro* functional analyses such as gene knockdown/overexpression, phenotype rescue assays, pathway interrogation, and cellular behavior studies. These investigations have collectively revealed a shared pathological basis underlying craniofacial malformations: disrupted developmental programs of CNCCs, and the central roles of key signaling pathways including FGF, Wnt, BMP/TGFβ, RA, and Shh. Furthermore, critical cellular processes such as EMT, directed migration, differentiation, and apoptosis are shown to be perturbed. The Xenopus model therefore provides robust *in vivo* evidence and a unique experimental platform for elucidating disease mechanisms, identifying pathogenic genes, and developing therapeutic strategies.

## 5 Translational applications and challenges in clinical implementation

The clinical translational potential of *Xenopus laevis* in the study of craniofacial developmental disorders is becoming increasingly evident, offering multiple avenues for application. Its remarkable self-regeneration capacity has elucidated cartilage-dependent repair mechanisms involving matrix metalloproteinases such as Mmp1 and Mmp13, highlighting the possibility of developing therapeutic strategies that do not rely on exogenous interventions (123). Through gene editing and embryological approaches, the *Xenopus* model has also clarified the central role of the RSPO2–RNF43/ZNRF3–WNT signaling axis in limb regeneration, offering key molecular targets and theoretical frameworks for regenerative medicine (124, 125). Additionally, serotonergic signaling has been shown to mediate structural repair, suggesting that neurotransmitter pathways may serve as novel therapeutic avenues (126). *Xenopus* efficiently screens teratogens (e.g., e-cigarette-derived vanillin), which disrupt retinoic acid signaling to induce malformations (127, 128). Some recent studies have used *Xenopus* oocytes to test new targets and identify new active drug candidates. Romero et al. developed the novel peptide RgIA4 through high-throughput screening of >200 synthetic analogs on α9α10 nAChR-expressing *Xenopus* oocytes, identifying a candidate with 100-fold higher potency than conventional analgesics for human and rodent targets (129).

Despite the unique advantages of *Xenopus* in the study of craniofacial developmental disorders, several limitations continue to hinder its translational applications. First, *Xenopus laevis* is an allotetraploid species (130), and its genomic complexity renders gene knockout and editing more technically challenging than in diploid models, thereby limiting precise genetic manipulation (131). Second, notable physiological and immunological differences exist between *Xenopus* and mammals, particularly the absence of placental and lactational systems, which constrains its ability to faithfully model certain human syndromes. Moreover, *Xenopus* is not suitable for investigating complex behavioral phenotypes or modeling chronic adult-onset diseases over extended periods, areas where murine models remain indispensable. Although *Xenopus* excels in elucidating developmental mechanisms, its findings must often be validated in human organoids, cell lines, or mammalian systems to ensure clinical relevance and translatability (132). Finally, the experimental toolkit for *Xenopus* remains underdeveloped; tissue-specific Cre-lox systems, comprehensive reporter gene libraries, and high-resolution imaging technologies are still lacking, which limits its utility for high-throughput screening and fine-scale mechanistic dissection.

## 6 Conclusion

In summary, *Xenopus* serves as a powerful model to investigate craniofacial development and associated disorders, offering insights into fundamental mechanisms and translational relevance. Future work should focus on integrating high-resolution single-cell and multi-omics approaches with *in vivo* functional assays in *Xenopus*, to systematically link candidate human variants to developmental mechanisms and accelerate the discovery of diagnostic and therapeutic targets for craniofacial anomalies.
